# Rheumatic autoimmune diseases in women and midlife health

**DOI:** 10.1186/s40695-015-0012-9

**Published:** 2015-12-02

**Authors:** Wendy Marder, Évelyne Vinet, Emily C. Somers

**Affiliations:** 1 Division of Rheumatology, Department of Internal Medicine, University of Michigan, Ann Arbor, MI USA; 2 Department of Obstetrics & Gynecology, University of Michigan, Ann Arbor, MI USA; 3 Division of Rheumatology, McGill University Health Centre, Montreal, Canada; 4 Division of Clinical Epidemiology, McGill University Health Centre, Montreal, Canada; 5 Department of Environmental Health Sciences, University of Michigan, 2800 Plymouth Rd, NCRC B14-G236, Ann Arbor, MI 48109-2800 USA

**Keywords:** Autoimmune diseases, Rheumatic diseases, Systemic lupus erythematosus, Rheumatoid arthritis, Scleroderma

## Abstract

Autoimmune diseases such as systemic lupus erythematosus (SLE), rheumatoid arthritis (RA), and systemic sclerosis (scleroderma) preferentially affect women, and are characterized by systemic inflammation leading to target organ dysfunction. The public health burden of autoimmune diseases, which collectively represent a leading cause of morbidity and mortality among women throughout adulthood, is substantial. While some features of these diseases have been observed to improve over the menopausal transition, such as disease flare rate in SLE and skin softening and thinning in scleroderma, others, such as swollen and tender joints and radiographically confirmed damage in RA may worsen. The general trends, however, are not consistent or conclusive for all disease-related manifestations. Of great importance is the recognition that comorbid diseases, including osteoporosis and accelerated cardiovascular disease, contribute excess morbidity and mortality that becomes increasingly apparent as women with autoimmune diseases undergo the menopausal transition.

## Background

Autoimmune diseases are characterized by systemic inflammation, in which a dysregulated immune system causes damage or dysfunction to target organs. Rheumatic autoimmune diseases include conditions such as systemic lupus erythematosus (SLE), rheumatoid arthritis (RA) and systemic sclerosis (scleroderma), in which the connective tissues (cartilage, joint synovium, skin) are most frequently targeted. Collectively, the autoimmune diseases are estimated to afflict over 7 % of the general population [1]; RA is among the most common autoimmune diseases, with RA prevalence of greater than 1 % of the adult female population in the United States [2]. While rheumatic autoimmune diseases can occur across the lifespan, the typical presentation occurs in mid- or late- adulthood [3]. These diseases are considerably more common in women than in men, with approximately 90 % of prevalent cases being female for SLE and scleroderma, and approximately 75 % for RA [3]. Effective targeted therapies for RA have rapidly expanded over the last decade, leading to improved outcomes, but treatment options for SLE and scleroderma remain largely based on traditional immunosuppressive and anti-inflammatory agents which are associated with a range of toxicities [4]. Major comorbidities for the rheumatic autoimmune diseases include premature cardiovascular disease and osteoporosis, due both to underlying disease and chronic exposure to glucocorticoids [5–7]. Data from the last 15 years have demonstrated that when autoimmune diseases are considered as a group, they rank among the 10 leading causes of death among women under age 75 years [8, 9].

While varying effects of estrogen and other sex hormones have been proposed related to the predisposition and development of autoimmune diseases and their comorbidities, the roles of genetics, environmental factors, and their interactions undoubtedly play significant roles [10]. This review will discuss the epidemiology and clinical features of three systemic rheumatic autoimmune diseases—SLE, RA and scleroderma—in relation to women in midlife.

## Review

This review is aimed at providing an overview of key topics related to women’s midlife health that are thought to have distinct features and implications for women with autoimmune diseases compared to the general population. While not intended to serve as an exhaustive review of the literature, the authors screened and reviewed studies predominantly from the last two decades related to epidemiologic patterns and clinical features of SLE, RA and scleroderma in relation to midlife, with emphasis on large, population-based studies when available, in order to synthesize recurrent themes.

### Epidemiologic overview

**Fig. 1:**
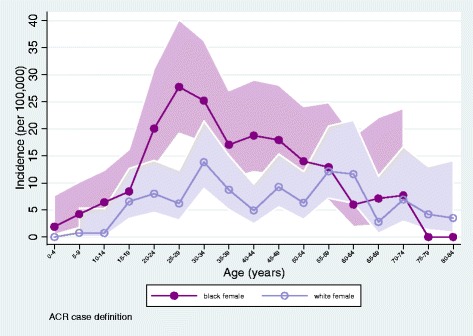
SLE incidence data from the Michigan Lupus Epidemiology & Surveillance (MILES) Program Registry [13]. SLE was defined according to the American College of Rheumatology (ACR) classification criteria for SLE [69, 70]

It is well-recognized that the majority of autoimmune diseases disproportionately afflict females. This is true for the rheumatic autoimmune diseases, with few exceptions, such as granulomatosis with polyangiitis (formerly termed Wegener’s granulomatosis) and vasculitis, which have sex ratios closer to 1:1 [3]. However, for the female-predominant diseases, the magnitude of female preponderance tends to wane in older age groups. In terms of lifestage of greatest risk, a long-held perception has been that SLE and other rheumatic disease are most likely to occur in women during their reproductive years. Recent epidemiologic data have provided the basis for a more nuanced view related to sex-specific patterns of disease, with SLE perhaps serving as the clearest example. A study of SLE incidence during the 1990s in the United Kingdom including 1638 incident cases revealed that among females in this population of predominantly European ancestry, risk of SLE rose steadily with age among females until 50–54 years of age, and thereafter steadily declined; in males, incidence increased steadily until 70–74 years of age before declining [11]. This data-driven observation of peak incidence in females occurring around the time of menopause was a novel finding, and contrasted with the premise of SLE as a “disease of women of childbearing age.” More recently, lupus registries from sociodemographically diverse populations in the United States have provided evidence for disparities in the risk of disease in different population subsets [12]. Data from the Michigan Lupus Epidemiology & Surveillance (MILES) Program [13] demonstrate a clear peak in incidence among black females during the 25–29 year age range; the magnitude and young age of peak incidence in this group are striking in comparison to other groups (Fig. 1). White females in the Michigan population experienced highest incidence between the ages of 30 and 34, but their peak was much less distinct and tapered slowly throughout the midlife years. With the younger risk of developing disease among black females, the highest prevalence occurred in midlife, at 40–44 years of age. MILES Registry data has also documented a higher SLE incidence and younger age of diagnosis in the Arab/Chaldean American population compared to non-Arab/Chaldean American whites, with prevalence among the Arab/Chaldean American population peaking in the 30–39 year age group [14].

Another registry in the United States, focused on the American Indian/Alaska Native population, found high rates of lupus, with incidence among American Indian/Alaska Native females similar to that of black females, and prevalence approximately 1.5 times higher [15]. Peak incidence in the American Indian/Alaska Native population (for females and males combined) occurred in the 40–49 year age span [15]. Together, contemporary SLE epidemiology data underscore varying patterns of risk in different population subsets, but clearly a high burden of disease among women in midlife.

### Primary ovarian insufficiency among women with rheumatic autoimmune diseases

When considering the subject of menopausal timing and its effects among populations of women with rheumatic autoimmune diseases, the possibility of exposure to gonadotoxic therapies used to treat severe manifestations of these conditions must be considered [16]. Potent first line immunosuppressive therapy for target-organ threatening manifestations of autoimmune diseases (e.g., lupus nephritis, systemic vasculitis, scleroderma) has historically been the combination of high dose corticosteroids and a gonadotoxic alkylating agent including cyclophosphamide, chlorambucil and even nitrogen mustard. Many women who received and continue to receive cyclophosphamide for rheumatic diseases, as well as for hematologic and solid organ malignancies, experience irreversible gonadal toxicity as a result of this therapy, leading to primary ovarian insufficiency [17–20]. Primary ovarian insufficiency, also known as premature ovarian failure, is defined as menopause before the age of 40 years [21]. Unlike with natural menopause, which occurs on average around age 50 years, 50 % of women with primary ovarian insufficiency still have variations in ovarian function and 5–10 % conceive following this diagnosis [22, 23]. There is lack of evidence that assisted reproductive technologies have efficacy in this population [23].

A major focus over the past two decades has been the development of alternate therapeutic regimens that minimize cyclophosphamide exposure in this patient population, as well as the use of adjuvant therapy such as with gonadotropin-releasing hormone analogues (e.g., leuprolide acetate), to preserve ovarian function during cyclophosphamide therapy [24]. Practice has evolved in recent years towards increased utilization of less toxic immunosuppressive therapies such as rituximab and mycophenolate mofetil in vasculitis and lupus nephritis respectively, as well as the adoption of lower-dose cyclophosphamide regimens (e.g., “Euro-Lupus” [25, 26]) for treating many cases of lupus nephritis. In addition to the increase in widespread off-label use of gonadotropin-releasing hormone analogues during cyclophosphamide therapy, these changes in standard of care therapy are allowing for rheumatic disease patients to achieve natural menopause at a rate that may soon mirror that of the general public.

### Systemic lupus erythematosus

SLE is a chronic, systemic inflammatory disease with a waxing and waning course, that can affect multiple organs, with the kidneys, joints, and skin most commonly involved. Symptoms can range from mild, including photosensitive rashes, mucosal ulcerations, and pleurisy, to severe and even life threatening, with rapidly progressive glomerular nephritis, central nervous system inflammation or hemolytic anemia. Lupus is classically considered an autoantibody-mediated disease, with evidence of immune complex deposition seen in renal and dermal biopsies of patients with active disease. Lupus patients also have heightened risk of cardiovascular disease that is not explained by traditional Framingham risk factors [27, 28], due at least in part to disease associated endothelial dysfunction that contributes to the accelerated atherosclerotic disease that also characterizes SLE [29–31]. Long-term studies of lupus outcomes have therefore included several means of quantifying disease. For example, the Systemic Lupus Erythematosus Disease Activity Index (SLEDAI) [32] is a widely used tool that measures recent disease activity, incorporating clinical history, physical exam and laboratory findings. The “SLICC/ACR” Damage Index [33] is used in lupus patients to quantify cumulative damage in 12 different target organs over time regardless of etiology; notably, it captures disease burden induced by cardiovascular disease, osteoporosis due to prolonged corticosteroid use and chronic inflammation, and the additive sequelae of premature menopause due to gonadotoxic therapy.

As stated previously, SLE incidence for women of predominantly European ancestry has been observed to rise steadily with age until the 50s, and decline thereafter. Multiple population based studies support the observation that the clinical course and disease manifestations of SLE are different if the disease onset occurs after natural menopause. Some studies have described a milder course associated with later disease onset, with general observations of less renal and mucocutaneous involvement, but higher rates of interstitial lung disease, neurologic complications and sicca symptoms [34–38]. Other studies have noted no difference in the clinical course of SLE between these two populations [39, 40]. However, data from a longitudinal and multiethnic cohort of 73 women with late-onset SLE (onset at age ≥ 50 years) and 144 matched women with earlier onset SLE found late-onset SLE to be independently associated with both damage (measured by the “SLICC/ACR” Damage Index [33]; OR 23.3, 95 % CI 4.0–141.6) and mortality (OR 10.7, 95 % CI 3.1–37.6) [37]. Thus, while in general it seems that most studies support a more mild course of lupus activity in later onset disease, the accrual of lupus damage as reflected in part by comorbid disease is probably greater in this group [41].

Varying degrees of improvement in lupus disease activity have been observed after women go through hormone withdrawal, whether due to natural menopause, hysterectomy or primary ovarian insufficiency after cyclophosphamide [41–44]. Although heterogeneous and somewhat difficult to compare given differences in age of the study populations and duration of follow-up, in general the research supports a trend toward fewer lupus disease flares after hormone withdrawal, but significantly more damage accrual.

### Rheumatoid arthritis

RA is a systemic disease that can lead to erosive, destructive joint damage, as well as “extra-articular” disease involving not just the joints, but target organ tissues of the body including the lungs, blood vessels, eyes and nervous system. This is particularly true for those people who are “seropositive” for the characteristic autoantibodies of rheumatoid factor and anti-cyclic citrullinated peptide antibodies. As mentioned above, RA is also associated with excess mortality compared with the general population, which is predominantly due to accelerated atherosclerotic cardiovascular disease [45–47]. As is true with antinuclear antibody positive diseases, RA preferentially affects women, in ratios around 4:1 [48], and has a peak incidence in females following menopause (age 55–64 years), a full two decades prior to age of peak incidence in men (75–84 years) [48]. Interestingly, the female-to-male incidence ratio after age 60 years is approximately 1:1, potentially implicating changes in sex hormones in the development of RA [49]. A disease with waxing and waning activity, RA is clearly influenced by sex hormones throughout adult life: multiple population-based studies have found that women with RA have a lower mean age at menopause compared to controls [50–52] and a pattern of RA symptom improvement or even remission during pregnancy is well recognized [50].

What is less well understood, however, is the role of gonadal hormones in both the risk for developing the disease, as well as the timing and severity of its manifestations. Observational studies that have assessed menopause by patient self-report, with variable lengths of recall, do not uniformly specify surgical or non-surgical menopause among their study populations, and have produced conflicting results. For example, a prospective cohort study of 31,336 women in Iowa, aged 55 to 69 years at cohort baseline and followed for 11 years, revealed 158 incident cases of RA, with age at last pregnancy and age at menopause each significantly inversely associated with RA [53]. In this study, no effect was found related to onset of menarche, oral contraceptive use or hysterectomy/oophorectomy, though those women who underwent later menopause (after age 51 years) were at decreased risk of developing RA compared to those who underwent menopause at or below age 45. However, a recent study, in which a group of 534 patients in a Canadian inception cohort of RA patients were divided into an “early menopause” (mean age 38.5 years) and “usual menopause” group (mean age 51.7 years), the age of RA disease onset was found to be similar between the groups [54]. Furthermore, the early menopause patients were more likely to be rheumatoid factor positive, a characteristic of the disease that is known to impart increased risk of erosive joint disease as well as extra-articular, systemic manifestations (e.g., vasculitis, interstitial lung disease). This study did include persons who underwent surgical menopause. In contrast, a subsequent study of 134 women with RA and earlier menopause (age <45 years) revealed that these women had more mild-moderate disease, and more rheumatoid factor negative disease, than women who underwent later menopause [52]; it is unclear if women who underwent surgical menopause were excluded from the earlier menopause group. It is therefore difficult to interpret these results other than to restate the observation that, like other autoimmune diseases, the role of sex hormones is significant but complex, and clearly not fully elucidated. However, general observations about the impact of menopause in this patient population are not in dispute. The heightened risk of accelerated cardiovascular disease [55], excessive bone density loss due to long-term corticosteroid therapy, and the possibility of primary ovarian insufficiency due to previous exposure to gonadotoxic therapies, in the context of ongoing underlying systemic inflammatory diseases, all confound the care and management of these patients as they age, and should prompt vigilance on the part of caregivers to address any modifiable risk factors associated with these conditions.

RA disease activity is typically measured using the 28-joint count disease activity scale (DAS-28) [56], an assessment of progression of bone X-ray changes, and patient-reported health assessment questionnaires. The largest study to address the effect of menopausal transition on RA was conducted in a cohort of early RA patients using these measures. Post-menopausal women in this cohort (n = 109) were found to have more significant joint damage on X-ray, in addition to higher scores on the DAS-28 and health assessment scores both at baseline and at 6-year follow-up, when compared to pre-menopausal women (n = 64) and age-matched men (n = 85) [51]. The menopausal state in this study was thought to be a primary factor underlying the differences observed between these groups of RA patients.

### Systemic sclerosis (or scleroderma)

Scleroderma is an inflammatory disease that causes vasculopathy and fibrosis and scarring in multiple target organs, including the vasculature, lungs, gastrointestinal tract and skin. Skin thickening is a defining feature of scleroderma, which is characterized by excessive production of extracellular matrix proteins (e.g., collagen, laminin, fibronectin) by skin fibroblasts [57]. The peak incidence of scleroderma is in the fifth and sixth decades, and scleroderma predominantly affects women (with a female-to-male ratios ranging from 3:1 to 14:1) [58].

In the general population, menopause is characterized by a low estrogenic state and is associated with skin thinning due to decreased extracellular matrix protein deposition by fibroblasts [59]. Although thinning of the dermis often accompanies aging, most studies suggest that collagen loss is more closely related to postmenopausal status than chronologic age, reflecting hormonal changes [60]. Investigators have observed a mean decline in dermal collagen of approximately 1–2 % per year after menopause [61]. Estrogen supplementation in postmenopausal women has been reported to improve skin thickness by increasing skin collagen content [62].

Although scleroderma most commonly occurs near the end of the reproductive period and predominantly affects women, there has been only one study investigating the impact of menopause on skin thickening in women with scleroderma [58]. Investigators, using previously collected data from 1070 women with scleroderma enrolled within the Canadian Scleroderma Research Group (CSRG) cohort, found that postmenopausal status in women with diffuse scleroderma was associated with a substantially lower mean modified Rodnan Skin Score, a validated measure of skin thickening, compared to premenopausal status (effect estimate of −2.62 units, 95 % CI −4.44, −0.80) [58]. This effect was independent of age, follow-up time, and disease duration. However, postmenopausal status had a smaller effect on skin thickening in women with limited scleroderma compared to women with diffuse scleroderma (effect estimate of −0.58 units, 95 % CI −1.50, 0.34).

The findings from this study are supported by previous experimental evidence showing that estrogen increases extracellular matrix protein production in skin fibroblast cultures of scleroderma patients [62]. In addition, an estrogen-receptor inhibitor (i.e., tamoxifen) induced a significant decrease of these extracellular matrix proteins in cultures of scleroderma skin fibroblasts [62]. Moreover, it is well-established that estrogen stimulates normal skin fibroblasts to produce transforming growth factor-beta 1, as well as monocytes and macrophages to produce platelet-derived growth factor, which are both key profibrotic cytokines in scleroderma skin disease [57, 63]. As scleroderma skin fibroblasts show increased expression of transforming growth factor-beta 1 receptor and platelet-derived growth factor receptor, the study investigators postulated that estrogen might play a role in scleroderma pathogenesis through its stimulatory effect on these two cytokines [58].

Furthermore, early menopause in women with scleroderma may contribute adverse lowering of bone mineral density in affected women, though the competing effects of underlying systemic inflammation and long term treatment with corticosteroids make it difficult to assess the relative impact of early menopause. Several studies have shown a potentially reduced bone mineral density in women with scleroderma compared to control women. A recent systematic literature review summarized data about the prevalence of low bone mineral density and its risk factors in scleroderma [64]. The search resulted in ten studies, which reported a lower bone mineral density in patients with scleroderma compared to matched controls, while two studies reported no difference. Potential risk factors for low bone mineral density in women with scleroderma included early age at menopause, as well as traditional risks factors such as family history of osteoporosis, age, low vitamin D levels, in addition to disease-related factors such as diffuse disease subtype, presence of internal organ involvement, and calcinosis. However, early menopause was inconsistently assessed across included studies, which makes it difficult to draw firm conclusions about the significance and size of its effect on bone mineral density in scleroderma women [64].

As mentioned previously, vasculopathy is an important disease-related manifestation in scleroderma. Since estrogen has well-established beneficial effects on the vascular system, the low estrogenic state associated with menopause has been suggested to aggravate vascular manifestations in scleroderma women [65]. In a retrospective cohort study of 189 scleroderma women with neither pulmonary arterial hypertension nor interstitial lung disease at baseline, investigators assessed the effect of postmenopausal status on the risk of isolated pulmonary arterial hypertension. During a mean follow-up of 15.9 years [standard deviation (SD) 11.3], 63 (33 %) women developed isolated pulmonary arterial hypertension. Postmenopausal status was significantly associated with more than a 5-fold increase in the risk of isolated pulmonary arterial hypertension, accounting for disease and human leukocyte antigen (HLA) subtypes [66]. Furthermore, another retrospective cohort study from the same group evaluated the effect of hormone replacement therapy on the risk of isolated pulmonary arterial hypertension in females with scleroderma [67]. Sixty-one postmenopausal women with limited scleroderma and without evidence of isolated pulmonary arterial hypertension or interstitial lung disease at cohort entry were studied. Among these, 23 were treated with hormone replacement therapy for a mean of 6.7 years (SD 3.7), of whom none developed isolated pulmonary arterial hypertension during follow-up. However, among the 41 women unexposed to hormone replacement therapy, 8 (20 %) developed isolated pulmonary arterial hypertension over a similar period of follow-up. This difference was not accounted for by age, autoantibody profile, lung diffusing capacity at menopause onset, or calcium channel blocker use. The investigators concluded that hormone replacement therapy might prevent the onset of isolated pulmonary arterial hypertension in patients with limited scleroderma.

Observational studies have also reported a higher prevalence of atherosclerotic cardiovascular disease in scleroderma patients in comparison to healthy individuals, with its presence being associated with poorer prognosis [68]. The mechanisms leading to increased atherosclerosis in scleroderma are not completely understood, but some have proposed endothelial dysfunction due to inflammation and vasculopathy, which might potentially interact with traditional risk factors, including age and menopause, as important contributing factors [68].

Although the menopause-related decline in estrogen appears to have a beneficial effect on skin thickening in scleroderma, it might have an adverse effect on the pulmonary arterial vasculature as well as atherosclerotic cardiovascular disease in affected women. Observational studies of menopause in women with scleroderma highlight the pleiotropic role that estrogen might play in scleroderma pathophysiology and prompt further research to better understand its complexity.

## Conclusions

It is difficult to disentangle the true impact of reproductive aging on the natural history of rheumatic autoimmune diseases. However, what seems clear is that the effects of major comorbidities associated with aging, which are likely accelerated in rheumatic autoimmune diseases, are magnified as these women approach midlife and beyond. This is due in part to the compounding effects of the underlying diseases and their treatments, such as long-term corticosteroid use and gonadotoxic immunosuppression, in addition to recognized vascular endothelial dysfunction that exists across the spectrum of these diseases. The public health burden of autoimmune diseases, which collectively represent a leading cause of morbidity and mortality among women throughout adulthood, is substantial. Their impact on women’s health becomes even more complex through menopause. With greater numbers of these patients achieving longer lives due to therapeutic advances, ascertaining the true impact of menopause on rheumatic autoimmune disease should be considered a priority for women’s health.
